# The Business Case for Simulation-based Hospital Design Testing; $90M Saved in Costs Avoided

**DOI:** 10.1097/pq9.0000000000000775

**Published:** 2024-11-15

**Authors:** Nora Colman, Christopher Chelette, Jayne Woodward, Misty Chambers, Kimberly Stanley, Sarah Walter, Vanessa Lampe Heimbuch, Caitlin Webster, Kiran Hebbar

**Affiliations:** * From the Division of Pediatric Critical Care, Department of Pediatrics, Children’s Healthcare of Atlanta, Atlanta, Ga.; † Department of Facilities Services, Children’s Healthcare of Atlanta, Atlanta, Ga.; ‡ Department of Facilities Services, Earl Swensson Associates, Inc., Nashville, Tenn.; § Page Southerland Page, Inc., Atlanta, Ga.; ¶ Nell Hodgson Woodruff School of Nursing, Emory University, Atlanta, Ga.

## Abstract

**Introduction:**

Simulation-based hospital design testing (SbHDT) applied during the design of a healthcare facility ensures that the architectural design supports safe, high-quality, and efficient care delivery beyond applicable building code compliance. This prospective investigation assesses the financial impact of SbHDT in the form of cost avoidance.

**Methods:**

In designing a new free-standing 400+ bed children’s hospital, SbHDT identified latent conditions early in the planning process to mitigate safety concerns related to the proposed design of 15 clinical areas. Architectural modifications were made to address concerns and resolve latent conditions before construction. The estimated cost of materials and labor to make an architectural change was documented for each architectural modification. Unit cost multiplied by unit count for each design element changed was summed together as total cost avoidance.

**Results:**

The cost to conduct the simulation was $1.6M (0.01% of overall project cost). Seven hundred twenty-two latent conditions were identified, and 57% of those latent conditions were mitigated by design changes. Ninety million dollars in costs were avoided by making design modifications before construction. Twenty-eight percent of latent conditions (n = 117) would have been cost-prohibitive to modify after construction.

**Conclusions:**

SbHDT harnessed evidence-based design to improve clinical care, optimize safety, and maximize investment. SbHDT was financially practical and had a significant impact on cost avoidance. Implementing SbHDT is associated with upfront costs, but long-term savings will accumulate over time through expenses avoided through mitigation of safety threats and operational savings.

## INTRODUCTION

The physical environment where people work is a critical component of a working healthcare system.^1^ Rigorous evidence-based design research undeniably demonstrates how the physical environment impacts healthcare outcomes.^2^ This set of guiding principles influences architectural decisions and strengthens the business case for building safer hospitals.^3–5^

Designing a hospital that supports the highest level of patient safety requires a collaborative and multidisciplinary approach that considers best practices and regulatory standards while balancing nuances in care delivery driven by local institutional culture and patient heterogeneity. A well-designed environment supports best practices, reduces variation in care through standardization, minimizes staff fatigue through optimal adjacency layout, reduces risk of contamination, minimizes environmental hazards, provides flexibility, and can adapt to varying staff and patient care needs.^6^

During the design of a healthcare facility, simulation-based hospital design testing (SbHDT) is a risk mitigation strategy that ensures that the architectural design supports safe, high-quality, and efficient care delivery.^7–10^ This rigorous and studied methodological approach harnesses evidence-based design as a translational force layered upon a human factors-based safety framework, Systems Engineering Initiative for Patient Safety (2.0), to improve clinical care and address safety risks.^1,8^ Due to competing priorities around managing construction costs and adhering to the building timeline, hospital executives may hesitate to incorporate simulation into the design process. Unfortunately, there is little evidence demonstrating the return on investment of this type of endeavor.^9^ Simulationists must justify investments for these projects and make a business case for executive leadership buy-in and project investment.

Cost avoidance in constructing a hospital refers to implementing measures during planning, design, and construction that prevent potential future expenses. Cost avoidance is money saved by not making design changes after a facility is constructed. The cost influence curve illustrates the relationship between cost and the ability to impact change over the life cycle of a facility project.^8,11^ As the design process progresses, additional changes become more expensive. If safety is not a focus of design, there may be a need for costly retrofitting and design enhancements to mitigate risk.^7,11^

SbHDT was used to evaluate the proposed architectural design of a new free-standing children’s hospital. This article extends previously published work and includes additional data from subsequent simulations.^8^ This prospective investigation assesses the financial impact of SbHDT. We hypothesized that applying SbHDT to the design process would avoid significant construction costs.

## METHODS

### Context

This new 1.5 billion (US) dollar children’s hospital was built to replace an aging landlocked facility. The facility is 19 stories, has 400+ beds, and spans 1.5 million square feet. This was an 8-year project from planning through construction. SbHDT was conducted in year three and took place in a “cardboard city” mockup built in a 100,000+ square foot warehouse in 2019.^8^

SbHDT is a robust systematic process improvement approach applied to proactively test the complex interface between people and the physical environment during the design process.^8^ This methodology includes simulated scenarios, Summarize, Anchor, Facilitate, Explore, Elicit (SAFEE) debriefing,^12^ and failure mode and effect analysis (FMEA).^13^ Our institutional review board granted this project a nonhuman subjects waiver.

SbHDT requires simulationists skilled in developing, facilitating, and debriefing simulations focused on systems integration and improvement science. Simulationists must comprehensively understand the design prototype being evaluated and the relationship between the built environment and safety.

### Interventions

SbHDT conceptual framework, rationale, project scope, methodology, and results are described in a previously published body of literature.^8,12,13^ SbHDT successfully identified potential latent conditions and mitigated risk related to the proposed schematic and detailed design of 15 distinct clinical areas. As staff implemented care processes, we identified latent conditions through interaction with specific design features that were either frequently used or high risk. High-risk design elements, defined in evidence-based design literature, are architectural components of the physical environment that are known to impact healthcare outcomes.^2,6,14^

**Fig. 1.**
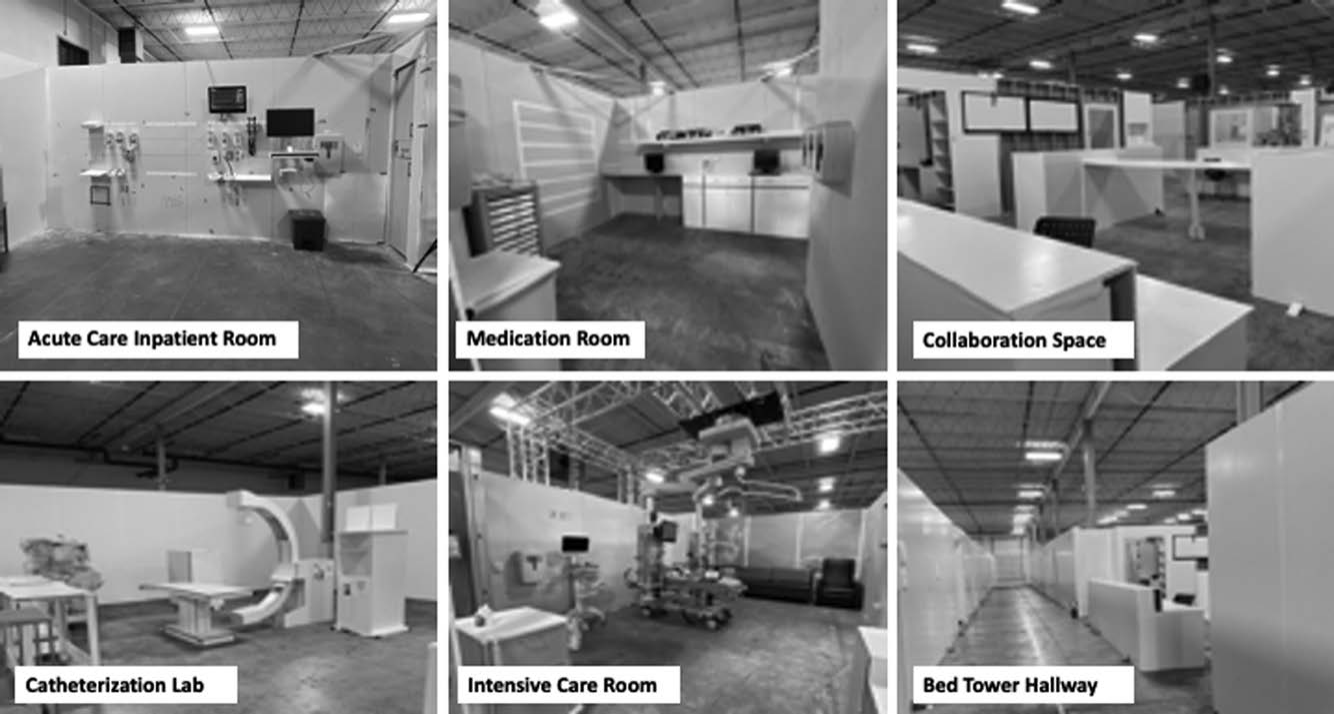
Photographs of cardboard city.

The cardboard mockup was built to scale and represent the unit’s configuration and layout. Mockup rooms included booms or head walls, built-in cabinetry, and wall-mounted fixtures. We staged the mockup with actual equipment and disposable supplies and if not available, were represented in cardboard (Fig. 1). Frontline staff (physicians, nurses, respiratory therapists, and patient care technicians) participated in the simulation. Executive leadership, departmental and service line leaders observed simulations. Clinical scenarios represented routine and high-risk care episodes. (**See Supplemental Digital Content 1, which describes the summary of SbHDT clinical scenarios,** http://links.lww.com/PQ9/A612.)

SAFEE debriefing guided clinical teams to identify potential latent conditions and active failures related to the architectural design. Each scenario was followed by a 45-minute debrief. The facilitator applied SAFEE, a five-step guide to identify latent conditions associated with the architectural design and explore potential active failures.^12^ During debriefing, a scribe documented each potential latent condition and associated active failure.

FMEA evaluated the impact of each latent condition identified during debriefing based on a process previously published.^13^ We used the FMEA rubric (Table 1) to score the severity and occurrence of each latent condition on a 5-point Likert scale. The four severity categories included: (1) patient and staff safety, (2) performance impact, (3) patient/family or staff experience, and (4) regulatory violation. The occurrence score defined the likelihood of a failure occurring. The two scores were multiplied together for a criticality score.^13^

**Table 1.** FMEA scoring rubric

	5	4	3	2	1
+ Severity	Catastrophic	Major	Moderate	Minor	No Harm
	Patient safety -Failure mode could result in permanent patient harm or death (SSE 1, SSE 2, SSE 3)	Patient safety -Failure mode could cause temporary patient harm and result in prolonged hospitalization SSE 4, SSE 5 -May lead to Hospital Acquired Condition (CLABSI, CAUTI, VAE, SSI, ADE, PIVIE, Fall, PUP, VTE) -Creates an environmental hazard (placement of equipment, furniture, connections, or cords that creates unnecessary crowding in patient care space)	Patient safety -Failure mode could result in the need for increased patient monitoring, treatment, and/or in intervention, but there is no patient harm. (precursor safety event) -Impacts the ability to provide safe care (according to organizational policy and procedure)	Patient safety -Failure mode reached patient but caused no harm (near miss event) -Impacts efficiency of delivered care (standardization, accessibility, and adaptability)	Patient safety -Failure mode did not reach the patient. -No impact to patient care
	Performance impact -Staff experiences lack of functionality. -Item completely fails to meet intended needs and performance is completely lost	Performance impact -Staff experiences a reduction in performance and productivity. -Failure can be overcome with modification, but there is some performance loss	Performance impact -Staff experiences a reduction in convenience. -Failure can be overcome with modification, but there is no performance loss	Performance impact -Staff experiences annoyance. -Failure can be overcome without modification or performance loss	Performance impact -Failure mode goes unnoticed by staff. -No need for modification, no loss in performance
	Employee safety -Failure mode results in loss of work for >90 d (long-term disability)	Employee safety -Design feature causes overexertion by requiring staff to kneel or squat -Failure mode results in loss of work for >8 d up to 90 d (short-term disability)	Employee safety -Design feature causes overexertion by requiring staff to work with the neck or back bent forward. -Failure mode results in loss of work for less than 8 d	Employee safety -Design feature causes overexertion by requiring staff to work with the hands above the head or elbows above the shoulder. -Failure mode results in missed work time during same shift	Employee safety -No ergonomic impact from utilizing design feature. -Failure mode did not result in any missed work
	Patient and family experience -Any grievance that requires referral to CMS	Patient and family experience -Any grievance that requires involvement of patient rep or review by patient safety and risk management	Patient and family experience -Any complaint or issue expressed to management that can be resolved promptly	Patient and family experience -Any complaint or issue expressed verbally to staff that can be resolved promptly.	Patient and family experience -No complaints or issues expressed
	Organizational priorities: -Cost: ≥$250,000 -Regulation risk: Immediate jeopardy finding: a situation in which the provider’s noncompliance with one or more requirements of participation has caused, or is likely to cause serious injury, harm, impairment, or death to a resident	Organizational priorities: -Cost: $100,000–$250,000 -Regulation risk: conditional finding: triggers a 90-d action to terminate the hospital’s Medicare Provider Agreement. Requires a credible allegation of compliance	Organizational priorities: -Cost: $10,000–$100,000 -Regulation risk: standard finding: requires an acceptable plan of correction for achieving compliance within a reasonable amount of time (usually within 60 d)	Organizational priorities: -Cost: <$10,000 -Regulation risk: failure mode reached patient but is not an accreditation/regulatory violation	Organizational priorities: -Cost: none -Regulation risk: no accreditation/regulatory violation
Occurrence	Frequent	Often	Sometimes	Occasionally	Seldom
Patient safety event	Likely to occur immediately or within a short period (weekly)	Probably will occur (monthly)	Possible to occur (quarterly)	Unlikely to occur (less than once a year)	Unlikely to occur in 1 to 3 y

Criticality Score is calculated by multiplying Severity score by Occurrence score: low priority (1–6), Medium priority (7–14), high priority (15–19), and very high priority (20–25).

Architectural: Potential threats involving room orientation, planned furniture location, fixed design elements, any mounted item, medical gases, and electrical outlets.

Clinical process: Potential threats related to ability to carry out policies and procedures.

Organizational operations: Potential threats involving resource allocation and management of supplies and equipment.

Technology: Potential threats related to monitoring, communication, and data storage.

ADE, adverse drug event; CAUTI, catheter-associated urinary tract infection; CLABSI, central line-associated bloodstream infection; CMS, Centers for Medicare and Medicaid Services; PIVIE, peripheral intravenous infiltration and extravasation; PUP, pressure injury prevention; SSE, serious safety event; SSI, Surgical Site Infection; VAE, ventilator-associated event; VTE, venous thromboembolism.

We conducted a single FMEA scoring session for each clinical area. The scoring team was comprised of executive leadership, departmental, and service line leaders. The scoring team assigned each latent condition to a severity category and determined the severity and occurrence score based on the FMEA rubric. The scoring team also subcategorized each latent condition as an architectural, clinical process, operational, or technological issue. A categorized and prioritized FMEA report was generated for each clinical area tested.

### Study of Intervention

SbHDT evaluated 2 sequential design planning phases: schematic design and design development. Clinical area layout, shape, and organization were evaluated in schematic design. In design development, SbHDT focused on designing specific room types such as inpatient/ intensive care unit rooms and operating rooms (ORs). Key design elements assessed during simulation are summarized in Table 2.

**Table 2.** Examples of Frequently Used and High-risk Design Elements Evaluated during Schematic and Detail Design Development

	Schematic Design*	Design Development†
Goals of design	• Minimize communication breakdown, minimize environmental hazards, standardize, improve visibility, minimize staff fatigue, reduce risk of injury, protect patient privacy, minimize sources of infection, provide safe, and efficient care delivery	• Optimize supply placement, optimize access to patient and equipment, optimize fixture, furniture, and equipment usability, improve visibility to patient
Frequently used design elements	• Location, orientation and adjacencies of medication room, clean supply room, soiled utility, equipment room • Overall design of workstations and collaboration spaces • Location of hand wash sinks and scrub sinks	• Wall-mounted fixtures; medical gases, electrical outlets, suction, temperature controls, diagnostic set (otoscope and ophthalmoscope), soap dispenser, glove box, paper towel holder, documentation stations, and lighting controls • Location of PPE
High-risk design elements	• Room layout • Unit layout • Orientation of critical adjacencies; imaging-emergency department, CICU-CVOR, call rooms-ICU • Hallway width, hallway alignment • Door sizes • Visibility; care team station layout, nursing alcove design and viewing windows	• Wall mounted fixtures: code blue button, sharps container, hand sanitizer, physiologic monitors • Design of millwork and cabinetry

* Schematic design: In this phase of design planning the layout, shape, and organization of clinical areas were evaluated.

† Design development: In this phase of design planning the design of specific room types [inpatient/ICU rooms, trauma and examination rooms in the emergency department, the general OR, and CVOR] were evaluated.

CICU, cardiac intensive care unit; CVOR, cardiovascular OR; ICU, intensive care unit; PPE, personal protective equipment.

In plan, do, study, act cycles, 2 rounds of testing for each clinical area for both schematic and design development were conducted to validate design modifications, assess the impact of design changes, and identify unexpected downstream consequences. Modifications made to the design were reflected in the mockup between the 2 rounds of testing to ensure the same level of fidelity.^8^ In the second round of testing, the same scenarios were repeated with the same degree of fidelity. Each potential latent condition identified in round 1 was rescored in round 2 using the same FMEA scoring process.^8,13^

Design modifications, solutions, and alternatives were up to the expertise of the architects. Design changes focused on mitigating latent conditions related to frequently used design elements and those with the highest threat to patient safety. Certain design elements, such as the square footage of clinical areas, were predetermined and not adjustable. However, design elements within that fixed space could be changed. We presented the simulation findings and all proposed changes to the hospital planning team (hospital executives and facilities leadership) for review and approval.

### Measures

After SbHDT, all reports from round 2 of simulation for both schematic and design development were combined into a master report. This master report described each potential latent condition in a single line item with its associated potential active failure and architectural modification (as applicable).

## ANALYSIS

The facilities and construction team reviewed the FMEA report. The estimated cost of materials and labor to make that architectural change following facility construction was documented for each architectural modification. We based the market value on labor and supply costs after the simulation in 2019. These costs were not projected at the time of construction (7 years postdesign planning) and, therefore, do not account for inflation. The cost of changing a single unit or design element and the unit count per clinical area were documented. This was multiplied together for a total cost per potential latent condition. If the same potential latent conditions were recurrent across multiple clinical areas, the cost of that architectural change was documented for each discrete area. For example, moving one code blue button was estimated to cost $2,500. For the cardiac intensive care unit, this was multiplied by 48 (number of cardiac intensive care unit beds) and by 60 (number of pediatric intensive care unit beds). Unit cost multiplied by unit count for each design element changed was summed together for total cost avoidance.

The master report was filtered once costs avoided were included. Latent conditions that were subcategorized as architectural issues and had a design modification with an associated avoidance cost were extracted into a consolidated FMEA report organized by clinical area. Each latent condition was also anchored to the FMEA criticality score.

We collected the warehouse lease, cost of supplies, and labor from the facilities department. The cost of personnel participation in simulation were collected from each of the clinical units’ directors. As our institution had a well-resourced simulation center, the cost of simulation staff, technicians, mannequins, supplies/equipment were already accounted for in our organization’s operational budget. Therefore, these expenses were not included in the analysis.

## RESULTS

**Fig. 2.**
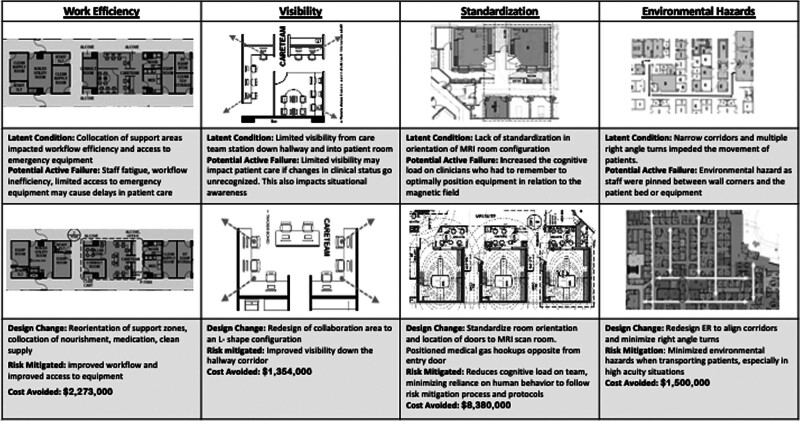
Examples of design changes avoiding over $1M in cost.

During SbHDT, 883 frontline staff participated in 146 scenarios conducted for 41 days of testing. The total cost to perform the simulation was $1,678,938 (Table 3), approximately 0.01% of the overall project cost. A total of 722 potential latent conditions related to architectural design were identified. Four hundred thirteen (57%) (median criticality score = 18) of potential latent conditions were mitigated with design changes. The remaining potential latent conditions were mitigated through process improvement, operational changes, or technology enhancements. Design modifications were associated with a cost avoidance of $91,142,200. Twenty-eight percent of latent conditions (n = 117) would have been cost-prohibitive (>$1 million) to modify after construction (Fig. 2). We detailed cost by area in Table 4.

**Table 3.** SbHDT Expenses

Expense	Cost
Staff participation	$238,162
Meals	$7,404
Mockup construction	$336,437
Warehouse furniture	$426
Warehouse cleaning	$7,805
Warehouse maintenance	$47,747
Warehouse utilities	$148,275
Warehouse lease	$892,682
Total cost	$1,678,938

**Table 4.** Summary of Design Modifications and Costs Avoided by Clinical Area

Clinical Area	No. latent conditions mitigated with design modifications		Median Criticality Score for Latent Conditions Mitigated with Design Modifications*	Summary and Examples of Design Modifications	Total Costs Avoided
		Bed tower			
Intensive care units: PICU, NICU, CICU	79		Median criticality score = 15	• Added scrub sink in alcove • Redesigned the collaboration space to improve visibility • Redesigned the provider workroom to provide more privacy • Redesigned the collaboration space layout to minimize traffic in front of the medication room corridor • Increased the size of respiratory equipment room and added a pass through • Increased the door size to the emergency equipment room • Upgraded the respiratory equipment room to include medical gas • Standardized call rooms and moved them closer to unit entrance • Relocated code blue button • Removed fixed documentation station • Added outlets to the footwall • Redesigned nurse server to accommodate a bedside cart • Repositioned paper towel, soap dispenser, and glove box	$20,293,200
General care; GPC, TICU, CSU, TSU, Heme/Onc	101		Median criticality score = 20	• Collocated nourishment/medication/clean supply • Redesigned the collaboration space to improve visibility • Added a pass-through to the clean supply room • Redesigned the chemotherapy medication room • Repositioned the code blue button • Reposited the otoscope/ophthalmoscope, soap dispenser, glove box, paper towel holder • Redesigned the nurse server to a 90-degree pass-through • Redesigned the in-room sink and cabinetry	$29,440,500
		Diagnostic and treatment areas			
Cardiac Operative Spaces; CVOR, cardiac catheterization laboratory, hybrid OR	62		Median criticality score = 20	• Redesigned the OR doors and increased door size of equipment room • Added windows to provide natural light • Widened hallway corridors and minimized right angle turns • Adjusted height of scrub sink • Repositioned door to clean core to improve flow • Increased size of window into the control room to 9 feet • Removed case work to provide space for sterile tables • Redesigned control room to minimize pinch points • Added and repositioned electrical outlets • Redesigned anesthesia casework	$8,776,600
Operative spaces: OR, postanesthesia care unit, day surgery	57		Median criticality score = 20	• Redesigned PACU to improve visibility • Added anesthesiologist workstations adjacent to control desk • Added glass to medication room and collaboration space to improve visibility in the PACU • Created three patient care zones that included medication room, clean supply, nourishment • Added a corridor to improve access and traffic to the ORs • Reconfigured the OR control desk • Increased size of the doorway to all ORs • Repositioned soiled utility adjacent to the AGV for improved access • Relocated entry doors to trauma operating room to minimize right angle turns and create a direct pathway from the trauma elevator into the OR • Relocated nourishment room closer to each end of the central core • Collocated the equipment room with soiled utility • Surgical bed rotated 90 degrees to improve flexibility for varying case types	$12,382,950
Emergency department	61		Median criticality score = 20	• Redesigned support areas and collocated nutrition, clean supply, and medication room • Redesigned hallways for direct pathways from examination rooms to trauma bays • Redesigned charge nurse for visibility to central access corridor, ambulance entry, holding area, and trauma pod • Redesigned corridors for more direct access to the CT scanner • Redesigned the medication preparation workspace • Adjusted height of medical gases • Reoriented the location of the door to exam rooms	$11,122,250
MRI	35		Median criticality score = 20	• Standardized room orientation and location of doors to MRI scan room • Positioned medical gasses opposite to the entry door • Revised position of medical gasses and casework • Adjusted locations of outlets and the physiologic monitor in the induction room • Repositioned gloves, paper towels, soap dispensers • Relocated code blue button • Reoriented windows in the control room for visibility to the bore of the scanner • Staggered windows behind the control rooms for privacy	$8,932,200
Interventional radiology	6		Median criticality score = 15	• Relocated documentation stations • Relocated lighting controls • Relocated anesthesia cabinets • Relocated code blue button	$168,000
Special care unit†	12		Median criticality score = 9	• Revised patient room layout to improve line of sight • Expanded donning area for adequate space for storage of paper scrubs, supplies, personal items, benches, mirrors, and personal donning/observing • Added a pass through between clean and dirty areas	$26,300
Total cost avoided					$91,142,000

* Criticality score = Severity × Occurrence; The FMEA rubric applied a 5-point Likert scale to score the severity and occurrence of each latent condition. The 4 severity categories included: (1) patient and staff safety, (2) performance impact, (3) patient/family or staff experience, and (4) regulatory violation. The occurrence score defined the likelihood of a failure occurring on a 5-point Likert scale. Criticality scores: low priority (1–6), medium priority (7–14), high priority (15–19), and very high priority (20–25).

† The special care unit is a clinical area designed for patients with highly infectious diseases (such as Ebola). This clinical area is infrequently occupied, therefore latent conditions were associated with a low occurrence score in comparison to the highly occupied areas of the hospital. This is reflected in a lower median criticality score, although severity scores still ranged from minor to catastrophic.

AGV, automated-guided vehicle; CICU, cardiac intensive care unit; CSU, cardiac stepdown unit; CVOR, cardiovascular OR; GPC, general pediatric care; Heme/Onc, hematology/oncology; NICU, neonatal intensive care unit; PICU, pediatric intensive care unit; PACU, postanesthesia care unit; TICU; technology dependent unit; TSU, transplant stepdown unit.

We estimate that several potential latent conditions could have been missed in the traditional design evaluation processes. For example, staff identified that the millwork inside of the patient room and the height of the nursing desk in the alcove blocked visibility to the head of the bed. Modifying the height of the desk alcove in all patient rooms avoided $340,000 in costs (Table 4).

Design modifications that would have cost over $1 million to retrofit postconstruction were made in the bed tower, magnetic resonance imaging (MRI), and emergency department (ED) (Fig. 2). The staff identified that the layout of patient beds and relation to support areas were inefficient. Nourishment, clean supply, and medication rooms were collocated to mitigate this latent condition. This created a “care zone” of patient rooms with associated support spaces. Care zones were standardized in the ED and bed tower to optimize workflow, avoiding over $2 million in cost.

Redesign of MRI mitigated safety threats related to equipment repositioning in the MRI scan room. When equipment such as ventilators/anesthesia machines are moved out of the way to bring patients into the scan room, they can become projectile objects if staff do not remember to relock them. Repositioning of the doors minimized the need to reposition equipment. Windows in the control room were oriented to provide direct visibility into the scanner’s bore for patient monitoring. Redesign of the MRI resuscitation bay improved accessibility if a patient needed to be emergently extracted from the MRI scanner. These modifications avoided more than $8 million in costs (Fig. 2).

Malalignment of hallways was an environmental hazard that impeded flow and safe patient transport in the ED. When the team maneuvered through the space with a patient stretcher and equipment, staff were pinned between wall corners, equipment, and the patient bed. To facilitate safer patient transport, hallway corridors were widened, right-angle turns were minimized, and corridors were redesigned to create a straight pathway for patient transport, avoiding $1.5M in costs (Fig. 2).

The ORs were reconfigured to optimize surgical bed positioning and provide more space for anesthesia at the head of the bed. This improved traffic flow so that providers could access the door to the clean core without bypassing the sterile surgical tables. Repositioning the entire ventilation system in each OR was necessary to meet air quality requirements. This avoided $19 million in costs.

Wall-mounted fixtures such as glove boxes, sharps containers, hand foam, soap, paper towel dispensers, and millwork (countertop surfaces) were design elements that frontline staff interacted with frequently. More than $14 million in cost was avoided by relocating mounted fixtures to optimize workflow efficiency. Minor renovations to millwork optimized the positioning of countertops, providing a clean workspace for medication preparation while maintaining adequate clearance from the sinks’ splash zone.

## DISCUSSION

This is the first article to describe cost avoidance when using SbHDT to inform the architectural design of a new hospital. Substantial and costly changes were made early because SbHDT offered a structured, robust, and safety-focused approach to analyzing design elements. SbHDT applied during design was optimally placed on the cost-influence curve^11^ where the long-term impact was high, and cost was low.^15^ We deliberately describe our findings in terms of cost avoidance and not return on investment because there is no universally accepted method for estimating the financial benefits of SbHDT.^9^

Williams et al^9^ recently described the financial impact of applying simulation-based clinical systems testing as a risk mitigation strategy before transitioning into a trauma facility. However, little evidence in the literature examines the financial impact of implementing simulation during the architectural design process. Hospital systems are often hesitant to use this type of project due to competing priorities around managing construction costs, adhering to the construction timeline, competing priorities for the hospital planning team, or bedside staff shortages.

One may still question why so many latent conditions required architectural modification? Healthcare delivery is highly variable based on local institutional culture, micro work system care processes, and patient heterogeneity. For this reason, a universal or standard design is unlikely to support the variation in care delivery across hospitals. SbHDT elucidates the realities and nuances of healthcare delivery so that the architectural design better aligns with local clinical practices and operations.

As part of the design process, it is important to make decisions that stick. SbHDT guides stakeholders in making decisions that focus on risk mitigation instead of reflecting preferences. SAFEE debriefing and FMEA scoring provided a strong rationale for making major design modifications because the impact of latent conditions had to be fully explored and expounded. The rationale was evident and justified when changes were presented to the hospital planning committee.

Although design modifications focused on addressing latent conditions with the highest threats to patient safety, those with low or medium criticality scores were also addressed. These latent conditions tended to fall under the performance impact (FMEA) category, which affected work efficiency and inconveniences for staff. Although many of these latent conditions could be tolerable, it is important that design supports the most efficient workflow and does not contribute to the development of future workarounds. These changes, such as repositioning wall-mounted fixtures early in the design process, were also cost-effective. After construction, these modifications would have high costs relative to the degree of impact.

In the second round of SbHDT testing, we ensured that design modifications did not have unintended downstream consequences that affected safety or efficiency. Simulation can be conducted in multiple rounds, leading to endless design iterations. This has the potential to hinder decision-making. We recommend limiting simulation to 2 rounds of testing to adhere to the construction timeline, schedule, and budget. Latent conditions that cannot be mitigated through design must be addressed through process and operational changes. Simulation-based clinical systems testing, used to evaluate the work system and care processes, can be applied longitudinally pre- and postoccupancy as part of transitioning into the new space.^7,16^

It is important that simulation is conducted on a near-final design prototype that has been developed and vetted by hospital leadership and clinicians. Because design modifications must be finalized before the onset of construction, we recommend conducting a simulation at the end of schematic and design development planning. From this point onward, design changes become costly and can significantly impact the project’s budget.

We believe that long-term return on investment is achieved through optimized operational efficiencies that impact ongoing costs over time. Long-term operational costs relate to inefficiencies, patient/staff safety events, regulatory violations, and lack of ability to adapt to changing patient care needs. When critical areas are designed to maximize efficiency and patient safety, future operational inefficiencies such as a need for increased staffing can be avoided. Hospital design that does not contribute to workarounds better supports safe practices, reducing adverse events and costs related to hospital-acquired conditions.^17,18^

### Challenges and Limitations

A universally accepted method for reporting financial impact in the form of return on investment and cost savings of SbHDT is needed. We can only demonstrate cost avoidance instead of cost savings because the hospital was not a functioning work system at the time of SbHDT implementation. Additionally, the impact of SbHDT on healthcare outcomes is too abstract to assess before the hospital is operational. Future postoccupancy research is needed to investigate the cost impact of SbHDT on reducing medical errors and optimizing operations. Due to limited resources, we did not account for inflation and fluctuating construction and labor costs expected to occur from design development to postoccupancy.

Architectural design is an iterative process where quality assurance and quality control measures are routinely applied during all design planning phases to identify architectural concerns, rectify errors, ensure that building standards are met, and that design aligns with clinical operations. Because our simulation was conducted early in the phases of schematic design and design development, it is possible that some of the latent conditions identified here would have been caught during the traditional design process, overestimating the costs avoided. A comparative analysis is needed to evaluate the role of simulation in identifying latent conditions versus the conventional design evaluation process.

Our institution is fortunate to have a robust simulation center with personnel skilled and trained in systems testing, debriefing, and FMEA. Large-scale simulation projects are expected to be part of our system-wide safety initiatives. Simulation staff salary, the cost of manikins, and equipment/supplies were already accounted for in our system’s operating budget. Therefore, these costs were not included in this study as an expense. Institutions without a robust simulation infrastructure will have an additional cost for simulation expertise, technicians, manikins, and equipment.

In addition to the monetary cost of staff participating in simulation, there is a nonmonetary impact, especially in the face of critical staffing shortages. During times of understaffing, it will be challenging for institutions to engage staff in simulation without compromising patient care. We would argue that a facility built without clinician feedback will likely not meet care delivery needs, contributing to further burnout, poor job satisfaction, and reduced retention.

## CONCLUDING SUMMARY

SbHDT utilized evidence-based design, a human factors-based safety framework (Systems Engineering Initiative for Patient Safety 2.0), and simulation to identify latent safety threats and generate solutions to improve clinical care, optimize safety, and maximize investment. SbHDT was financially practical and had a significant impact on cost avoidance. There is an up-front cost to conduct SbHDT. Still, long-term savings will incrementally accumulate over the life cycle of the hospital in the form of expenses avoided through the mitigation of safety threats and operational savings.

## ACKNOWLEDGMENTS

The authors would like to acknowledge the Children’s Healthcare of Atlanta Simulation Center who have done work to pave the way for this publication.

## Supplementary Material



## References

[1] Carayon P Schoofs Hundt A Karsh BT. Work system design for patient safety: the SEIPS model. Qual Saf Health Care. 2006;15(Suppl 1):i50–i58. 17142610 10.1136/qshc.2005.015842 PMC2464868

[2] Joseph A Rashid M. The architecture of safety: hospital design. Curr Opin Crit Care. 2007;13:714–719. 17975396 10.1097/MCC.0b013e3282f1be6e

[3] Sadler BL DuBose J Zimring C. The business case for building better hospitals through evidence-based design. HERD. 2008;1:22–39. 21161906 10.1177/193758670800100304

[4] Geis GL Pio B Pendergrass TL. Simulation to assess the safety of new healthcare teams and new facilities. Simul Healthc. 2011;6:125–133. 21383646 10.1097/SIH.0b013e31820dff30

[5] Peavey EK Zoss J Watkins N. Simulation and mock-up research methods to enhance design decision making. HERD. 2012;5:133–144. 23002575 10.1177/193758671200500313

[6] Joseph A. Designing for patient safety: developing methods to integrate patient safety concerns in the design process 2012. Available at https://www.healthdesign.org/sites/default/files/chd416_ahrqreport_final.pdf. Accessed June 2024.

[7] Colman N Doughty C Arnold J. Simulation-based clinical systems testing for healthcare spaces: from intake through implementation. Adv Simul (London, England). 2019;4:19. 10.1186/s41077-019-0108-7 PMC6676572 31388455

[8] Colman N Edmond MB Dalpiaz A. Designing for patient safety and efficiency: simulation-based hospital design testing. HERD. 2020;13:68–80. 10.1177/1937586720921777 32367742

[9] Williams SA Fitzpatrick K Chandler NM. Financial and safety impact of simulation-based clinical systems testing on pediatric trauma center transitions. Pediatr Qual Saf. 2022;7:e578. 36032192 10.1097/pq9.0000000000000578 PMC9416763

[10] Lawrence JF Tsang R Fedee G. Prevention of latent safety threats: a quality improvement project to mobilize a portable CT. Pediatr Qual Saf. 2021;6:e422. 34235351 10.1097/pq9.0000000000000422 PMC8225372

[11] Taylor E Hignett S Joseph A. The environment of safe care: considering building design as one facet of safety. In: International Symposium on Human Factors and Ergonomics in Healthcare: Advancing the Cause. 2014;3:123–127.

[12] Colman N Dalpiaz A Walter S. SAFEE: a debriefing tool to identify latent conditions in simulation-based hospital design testing. Adv Simul (London, England). 2020;5:14. 10.1186/s41077-020-00132-2 PMC7384892 32733695

[13] Colman N Stone K Arnold J. Prevent safety threats in new construction through integration of simulation and FMEA. Pediatr Qual Saf. 2019;4:e189. 31572890 10.1097/pq9.0000000000000189 PMC6708643

[14] Ulrich RS Zimring C Zhu X. A review of the research literature on evidence-based healthcare design. HERD. 2008;1:61–125. 21161908 10.1177/193758670800100306

[15] Wingler D Machry H Bayramzadeh S. Comparing the effectiveness of four different design media in communicating desired performance outcomes with clinical end users. HERD. 2019;12:87–99. 10.1177/1937586718796626 30165754

[16] Colman N Hebbar K. Simulation enhances safety evaluation in the design of new healthcare facilities. Curr Treat Options Peds. 2020;6:214–225.

[17] Ventre KM Barry JS Davis D. Using in situ simulation to evaluate operational readiness of a children’s hospital-based obstetrics unit. Simul Healthc. 2014;9:102–111. 24401917 10.1097/SIH.0000000000000005

[18] Halbesleben JR Wakefield DS Wakefield BJ. Work-arounds in health care settings: Literature review and research agenda. Health Care Manage Rev. 2008;33:2–12. 18091439 10.1097/01.HMR.0000304495.95522.ca

