# Supplementary material for: The Business Case for Simulation-based Hospital Design Testing; $90M Saved in Costs Avoided
Source: Pediatr Qual Saf. 2024 Nov 15;9(6):e775. doi: 10.1097/pq9.0000000000000775 (PMC11567707; doi:10.1097/pq9.0000000000000775)
Supplement: Supplementary file 1 [file pqs-9-e775-s001.pdf]

Clinical Area	Examples of clinical scenarios
Intensive Care Unit; Pediatric, Neonatal, Cardiac Intensive Care Unit	• 6-month-old with respiratory failure requiring intubation<br>• 6-month-old with respiratory failure requires cannulation to ECMO<br>• Infant with Hypoplastic Left Heart Syndrome admitted, requires intubation and central line placement<br>• Adolescent patient with myocarditis has a cardiac arrest and is cannulated to ECMO
General Pediatric Care<br>Technology Intermediate Care Unit, Transplant Stepdown Unit, Hematology/Oncology	• 11-month-old with patient with bronchiolitis requiring high flow nasal cannula decompensates and a rapid response is called<br>• 10-year-old patient receiving standard tracheostomy care develops tracheostomy obstruction requiring tracheostomy exchange<br>• 11-month-old patient with history of congestive heart failure is admitted with bronchiolitis develops respiratory distress<br>• 14-year-old female with low cardiac output syndrome secondary to dilated cardiomyopathy on a continuous milrinone infusion who has an arrythmia and requires synchronized cardioversion<br>• Patient with peritonitis requiring peritoneal dialysis<br>• A 6-year-old patient with leukemia and hemolytic anemia receives standard care and then develops blood transfusion reaction to blood requiring hemodynamic support
MRI	• Patient requiring sedation with general anesthesia<br>• Patient required sedation that develops laryngospasm that develops cardiac arrest
Operating room/PACU/Special Procedures, Day surgery, Catheterization Lab/Cardiovascular Operating Room	• 2-year-old patient undergoes tonsillectomy and adenoidectomy with bronchoscopy. Patient develops airway bleeding requiring stabilization.<br>• 14-year-old patient undergoes a laparoscopic gall bladder removal<br>• 5-year-old patient with cystic adenomatoid malformation undergoes bronchoscopy, laryngoscopy, and endoscopy. Patient has a bleeding ulcer requiring intervention<br>• Infant with Hypoplastic Left Heart Syndrome undergoes Norwood procedure<br>• 10-day old patient with Hypoplastic Left Heart Syndrome undergoes pulmonary artery band and stent in the hybrid catheterization lab
Emergency Department (ED)	• 5-year-old child requires laceration repair, develops laryngospasm during sedation requiring intubation<br>• 3-year-old child following a motor vehicle accident with blunt abdominal trauma<br>• 3-week-old infant with fever who requires a septic work up

Supplemental Digital Content 1: Examples of SbHDT clinical scenarios

ECMO: Extracorporeal Membrane Oxygenation, PACU: Post Anesthesia Recovery Unit
